# Unique transcriptomic alterations in 5XFAD;PS19 mouse model identify glial lipid dysregulation and coordinated microglial–oligodendrocyte responses

**DOI:** 10.1002/alz.71742

**Published:** 2026-08-10

**Authors:** Jung Hyun Park, Byungwook Kim, Md Mamun Al‐Amin, Mason Douglas Tate, Ahmad Daniel Sharify, Sutha K. John, Hande Karahan, Hui‐Chen Lu, Luke Child Dabin, Jungsu Kim

**Affiliations:** ^1^ Stark Neurosciences Research Institute Indiana University School of Medicine Indianapolis Indiana USA; ^2^ Department of Medical and Molecular Genetics Indiana University School of Medicine Indianapolis Indiana USA; ^3^ Program in Neuroscience Indiana University Bloomington Indiana USA; ^4^ The Gill Institute for Neuroscience Indiana University Bloomington Indiana USA; ^5^ Medical Neuroscience Graduate Program Indiana University School of Medicine Indianapolis Indiana USA; ^6^ Department of Psychological and Brain Sciences Indiana University Bloomington Indiana USA; ^7^ Department of Biostatistics & Health Data Science Indiana University School of Medicine Indianapolis Indiana USA

**Keywords:** 5XFAD;PS19 mouse model, Accelerating Medicines Partnership Program for Alzheimer's Disease, Alzheimer's disease, amyloid beta, cross‐species alignment, neurodegeneration, single‐nucleus RNA sequencing, tau, transcriptome

## Abstract

**INTRODUCTION:**

Alzheimer's disease (AD) features amyloid beta (Aβ) plaques and tau tangles, yet how their coexistence reshapes brain transcriptomic programs remains unclear.

**METHODS:**

We performed high‐quality, sex‐balanced single‐nucleus RNA sequencing of 5XFAD (Aβ), PS19 (tau), and combined 5XFAD;PS19 mice.

**RESULTS:**

We identified transcriptional programs that emerged most prominently under combined pathology. These programs included disruption of glial lipid metabolism and immune pathways at the network level, alongside immune and synaptic alterations coordinated between microglia and oligodendrocytes. Cross‐species analyses further revealed that the pathway‐level alterations under combined pathology, particularly in immune, lipid, and cell cycle programs, exhibited the strongest concordance with human AD datasets, underscoring their translational relevance.

**DISCUSSION:**

Beyond benchmarking mouse models, this study provides a high‐quality transcriptomic resource to dissect multicellular disease mechanisms in AD and to prioritize therapeutic targets for a network‐level systems pharmacology approach.

## Highlights

- A high‐quality single‐nucleus RNA sequencing resource was generated from 5XFAD (amyloid beta [Aβ]), PS19 (tau), and 5XFAD;PS19 mice.
- Lipid metabolism and immune networks are disrupted in an Aβ–tau co‐pathology model.
- Immune and synaptic alterations were coordinated between microglia and oligodendrocytes.
- 5XFAD;PS19 data best aligns with human Alzheimer's disease data across immune, lipid, and cell‐cycle pathways.

## 1 BACKGROUND

Alzheimer's disease (AD) is a progressive neurodegenerative disorder characterized by two core biomarkers: amyloid beta (Aβ) plaques and tau neurofibrillary tangles.^1^, ^2^, ^3^ A growing body of evidence highlights the critical interplay between these pathologies, potentially through synapses and glial activation, that drives disease progression.^4^, ^5^, ^6^, ^7^, ^8^ In line with this, Busche and Hyman^4^ argued that anti‐Aβ therapies alone may offer limited benefit once symptoms emerge, underscoring the need to consider the synergistic relationship between Aβ and tau when developing therapeutics. However, most preclinical studies have examined Aβ and tau in isolation, using either Aβ amyloidosis models that develop robust Aβ deposition without tau pathology, or tauopathy models that accumulate hyperphosphorylated tau in the absence of Aβ.^9^, ^10^ Some of the most widely used mouse models include the 5XFAD amyloid mouse model overexpressing mutant human amyloid precursor protein (*APP*) and presenilin 1 (*PSEN1*) transgenes and the PS19 Tau model overexpressing P301S mutant human microtubule‐associated protein tau (*MAPT*).^11^, ^12^ While single‐pathology models have provided invaluable insights into the biology of Aβ or tau alone, they inherently do not capture the interactions between Aβ and tau pathologies that occur in human AD brains. In line with this limitation, human longitudinal studies show that a markedly increased risk of progression from mild cognitive impairment to AD is largely confined to individuals with concurrent Aβ and tau abnormalities, rather than those with only Aβ or only tau pathology.^13^ These findings highlight the need for preclinical models that incorporate both Aβ and tau to understand how these pathologies interact to shape molecular and cellular changes in AD.

Recent advances in single‐cell and single‐nucleus RNA sequencing (sc/snRNA‐seq) have transformed understanding of AD by resolving transcriptional changes at single‐cell resolution.^14^, ^15^ These studies have identified multiple disease‐associated glial subtypes with distinct transcriptional states and selectively vulnerable neuronal populations in amyloidosis models^16^, ^17^, ^18^ or tauopathy models.^19^, ^20^, ^21^ However, it remains unclear how the co‐occurrence of Aβ and tau drives transcriptional changes across different cell types, both in the expression level of individual genes and coordinated gene networks. Addressing this important question requires sc/snRNA‐seq studies with sufficient cell numbers from multiple biological replicates, balanced sex representation, and rigorous preprocessing to ensure robust and biologically interpretable single‐cell inferences. Moreover, direct transcriptomic alignment of mouse models with human AD signatures, vital for translational modeling, has rarely been demonstrated previously. Bridging these gaps is critical, as models that faithfully recapitulate the molecular complexity of human AD can enable mechanistic insight and guide precise therapeutic discovery.

In this resource study, we performed sex‐balanced snRNA‐seq experiments with 5XFAD, PS19, and 5XFAD;PS19 mice alongside wild‐type (WT) controls to investigate how combined Aβ and tau pathologies reshape cell type–specific transcriptomics relative to each single pathology. Our machine‐assisted nuclei isolation protocol minimizes ambient RNA contamination^22^ and enables the capture of large numbers of high‐quality nuclei across diverse cell types. Using high‐dimensional weighted gene co‐expression network analysis (hdWGCNA),^23^ we identified synaptic, immune, and lipid‐metabolism modules that demonstrated the most pronounced alterations in the combined 5XFAD;PS19 model. Microglial and oligodendrocyte clusters associated with the immune module consistently emerged as key contributors, exhibiting synaptic suppression in differential expression analyses and immune modulation inferred by CellChat.^24^ Cross‐species comparisons further revealed that the 5XFAD;PS19 model exhibits markedly stronger transcriptomic concordance with human AD than either single‐pathology model alone. Together, this study clarifies how Aβ and tau interact at the transcriptomic level, contextualizing findings from single‐pathology models. To further support the research community, we provide this snRNA‐seq dataset as a publicly available resource that can be queried to examine cell type–specific transcriptomic changes attributable to Aβ, tau, or their interaction and to support hypothesis generation in the context of multi‐pathology transcriptomics.

## 2 METHODS

### 2.1 Animals

To generate our experimental mouse models, we crossbred hemizygous 5XFAD mice [B6SJL‐Tg(APPSwFlLon, PSEN1*M146L*L286V)6799Vas/Mmjax; #034840],^11^ which model Aβ amyloidosis, with hemizygous PS19 mice [B6.Cg‐Tg(Prnp‐MAPT*P301S)PS19Vle/J; #024841],^12^ which model tauopathy. This breeding strategy generated four littermate genotypes: WT, 5XFAD (Aβ amyloidosis), PS19 (tauopathy), and the double‐transgenic 5XFAD;PS19 (combined pathology). All animals were housed under standard laboratory conditions with unrestricted access to food and water at the Stark Neuroscience Research Institute. All experiments were approved by the Institutional Animal Care and Use Committee at Indiana University and performed in accordance with institutional guidelines (IACUC protocol #24125).

### 2.2 Tissue collection

Mice were anesthetized via the intraperitoneal injection of Avertin (250 mg/kg tribromoethanol; Sigma−Aldrich), followed by transcardial perfusion with ice‐cold 1x phosphate‐buffered saline. The brains were dissected immediately, and the anterior and posterior cortices were isolated, flash‐frozen on dry ice, and stored at −80°C. The posterior cortex was gently ground in 1X Tris‐buffered saline (TBS) containing protease and phosphatase inhibitors (Roche), and then centrifuged at 20,000 × g for 20 minutes at 4°C. The supernatants were transferred to new tubes, and the pellets were resuspended in 1X TBS with inhibitors, followed by sonication to extract soluble intracellular proteins. The homogenates were subsequently centrifuged at 20,000 × g for 20 minutes at 4°C. The supernatants were collected into new tubes, and the final pellet was further extracted in 5 M guanidine hydrochloride to extract insoluble proteins, using a rotator at room temperature for 3 hours.

### 2.3 Meso Scale Discovery assay for Aβ and tau

The quantification of the Aβ and tau proteins was performed using electrochemiluminescence‐based Meso Scale Discovery (MSD) assays, as previously described.^25^, ^26^ Briefly, Aβ concentrations were measured in guanidine‐soluble cortical fractions using the V‐PLEX Plus Aβ Peptide Panel 1 (6E10) Kit (MSD; K15200E). The levels of phosphorylated tau (Thr231) and total tau were measured from the sonicated TBS fractions using the Phospho (Thr231)/Total Tau Kit (MSD; K15121D). All procedures followed the manufacturer's protocols and were analyzed on a MESO QuickPlex SQ 120 multiplex imager.

### 2.4 Machine‐assisted nuclei isolation

Nuclei were isolated via the Singulator 100 system (S2 Genomics) as described previously,^22^ with all steps performed on ice. Briefly, frozen tissue was processed in pre‐chilled nucleus isolation cartridges with RNase inhibitor. The resulting nuclear suspension was pelleted by centrifugation, and the pellet was then purified through density gradient centrifugation using a 20% Nuclei Debris Removal Stock Solution in Nuclear Storage Reagent to remove myelin and other debris. After a final wash and centrifugation step, the purified nuclei were resuspended in resuspension buffer (2% bovine serum albumin in Nuclear Storage Reagent) for downstream applications.

**RESEARCH IN CONTEXT**

1. **Systematic review**: We reviewed the literature on transcriptomic studies in Alzheimer's disease (AD) mouse models and human *post mortem* datasets using PubMed. Prior studies identified disease‐associated glial subtypes and vulnerable neuronal populations in amyloidosis or tauopathy models separately. However, it has remained unclear how amyloid beta (Aβ) and tau co‐occurrence reshapes transcriptional programs across cell types and whether combined‐pathology models align with human AD signatures.
2. **Interpretation**: Two transcriptional programs emerged with combined Aβ and tau pathology: glial lipid metabolic dysregulation and coordinated microglial–oligodendrocyte responses involving immune and synaptic alterations. Both pathways were less apparent in single‐pathology models. The combined model showed the strongest pathway‐level concordance with human AD, particularly across immune, lipid, and cell cycle programs.
3. **Future directions**: Our publicly available high‐quality single‐nucleus RNA sequencing dataset enables the research community to generate new hypotheses based on more AD‐relevant double pathology transcriptomics. Furthermore, systematic integration of mouse–human concordant pathways, including the Accelerating Medicines Partnership Program for Alzheimer's Disease alignment, may help prioritize conserved druggable nodes for network‐level therapeutic development.

### 2.5 snRNA‐seq library preparation and sequencing

Purified nuclei were stained with trypan blue and manually counted using a disposable hemocytometer (C‐Chip, SKC, Inc.) under a 40x objective on an EVOS XL Core microscope. Each suspension was adjusted to 1000 nuclei/µL and loaded onto a Chromium Chip G for GEM (gel bead‐in‐emulsion) generation and barcoding using the Chromium Controller (10x Genomics). Library preparation followed the manufacturer's protocol using the Chromium Next GEM Single Cell 3′ v3.1 Dual Index kit (10x Genomics) with SPRIselect magnetic bead purification (Beckman Coulter Life Sciences). cDNA and library integrity were evaluated using an Agilent 2100 Bioanalyzer using the High Sensitivity DNA Kit (Agilent Technologies), and concentrations were measured using a Qubit Fluorometer with the dsDNA High Sensitivity Assay Kit (Thermo Fisher Scientific). Libraries were sequenced on an Illumina NovaSeq 6000 (v1.5 S2 flow cell) using a 28‐10‐10‐91 read configuration.

### 2.6 snRNA‐seq data analysis

#### 2.6.1 Data processing

**FIGURE 1:**
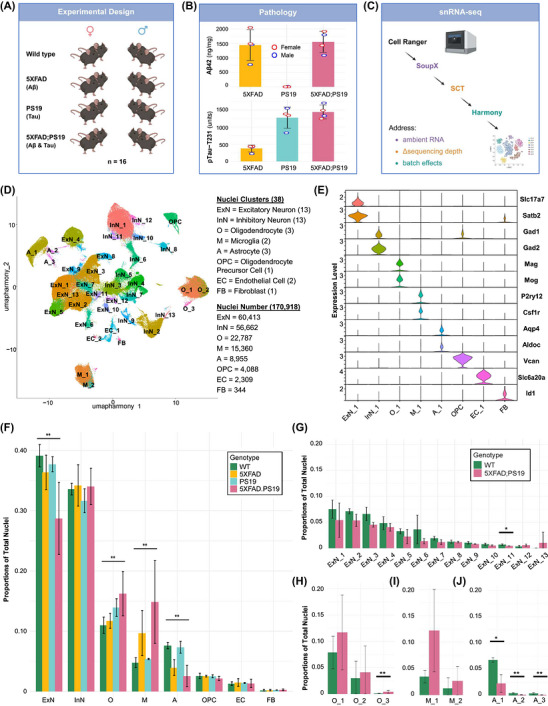
snRNA‐seq identifies differences in cell proportions across mouse models of Aβ amyloidosis, tauopathy, and their combined pathology. A, Experimental design. Cortical tissue from two mice per sex per genotype (*n* = 16) was used to generate snRNA‐seq data. B, The levels of insoluble Aβ42 (above) and phosphorylated tau (Thr231) were measured from the cortex of 5XFAD, PS19, and 5XFAD;PS19 mice, using Meso Scale Discovery (MSD) electrochemiluminescence assay. Each dot represents an individual sample (red = female, blue = male). Error bars denote standard deviation. C, Workflow of snRNA‐seq data preprocessing. Isolated nuclei were processed using CellRanger, followed by ambient RNA removal with SoupX. We then adjusted for sequencing depth per nucleus using SCTransform and integrated all 16 samples, correcting for experimental and technical batch effects with Harmony. D, UMAP visualization of 170,918 nuclei across 38 annotated clusters: excitatory neurons (ExN_1 to ExN_13), inhibitory neurons (InN_1 to InN_13), oligodendrocytes (O_1 to O_3), microglia (M_1 to M_2), astrocytes (A_1 to A_3), oligodendrocyte precursor cells (OPC), endothelial cells (EC_1 to EC_2), and fibroblasts (FB). E, Stacked violin plot showing scaled expression of key marker genes for the largest cluster within each cell type. F, Proportions of total nuclei per cell type across the four genotypes. G–J, Cluster‐level proportions of total nuclei between WT and 5XFAD;PS19 mice in excitatory neurons (G), oligodendrocytes (H), microglia (I), and astrocytes (J). Nuclei proportions were analyzed using the propeller function in the speckle R package. Error bars represent standard deviation (*P < 0.05, **P < 0.01). Aβ, amyloid beta; snRNA‐seq, single‐nucleus RNA sequencing; UMAP, uniform manifold approximation and projection; WT, wild type

The sequencing data were processed using the Cell Ranger pipeline (v7.2.0, 10x Genomics) and aligned to the mouse reference genome GRCm38 (gex‐mm10‐2020‐A).^27^ The filtered feature‐cell barcode matrices (including the hashtag count matrix) generated by CellRanger were loaded in RStudio (2023.11.999) running R (v4.2.1). SoupX (v1.6.2)^28^ was used to estimate and correct for ambient RNA contamination using default settings. Doublets were identified using DoubletFinder (v2.0.4). The cleaned data were analyzed using Seurat (v5.1.0). For quality control, nuclei with > 1% mitochondrial reads or < 200 unique reads were excluded from analysis. The data were normalized via SCTransform (v0.4.1),^29^ and the 16 samples were merged based on 3000 variable features. Integration was performed using Harmony,^30^ with genotype and sex included as covariates to correct for batch effects. After preprocessing, a total of 170,918 high‐quality nuclei were retained for downstream analysis. Figures 1A and 1C were created using https://BioRender.com.

#### 2.6.2 Clustering and annotation

Non‐linear dimensionality reduction was performed by running uniform manifold approximation and projection (UMAP) on the first 30 principal components. Clustering was performed using the FindNeighbors and FindClusters functions, and multiple resolutions (0.1–1.2) were evaluated for consistency and stability using clustree (v0.5.1).^31^ A resolution of 0.6 was selected for downstream analysis. The marker genes for each cluster were identified using the FindAllMarkers function. Clusters were annotated via scMCA (v0.2.0),^32^ PanglaoDB,^33^ and Semi‐Automated Hand Annotation (SAHA, v0.9.1).

#### 2.6.3 Cell type proportion analysis

The cell type proportions were quantified using speckle (v0.0.3).^34^ Proportions were calculated using the getTransformedProps function with logit transformation, applied separately for cell types and clusters. Group comparisons between 5XFAD, PS19, and 5XFAD;PS19 versus WT were performed separately using the propeller.ttest function.

#### 2.6.4 Gene co‐expression network analysis with hdWGCNA

hdWGCNA (v0.3.03)^23^ was used to identify co‐expression modules. The analysis was performed using the RNA assay, with metacells constructed by aggregating nuclei by cluster and biological sample (*k* = 35) to reduce data sparsity. Normalized metacell expression matrices were used to construct co‐expression networks with a soft‐threshold power of 7, as determined by TestSoftPowers. Co‐expression modules were then identified based on pairwise gene expression correlations and hierarchical clustering of the topological overlap matrix. Module eigengenes (hMEs) were calculated across the entire integrated snRNA‐seq dataset using Harmony for batch correction. Module eigengene‐based connectivity (kME) scores were computed to identify hub genes within each module. Genotype‐specific perturbations in network activity were evaluated via differential module eigengene analysis using the FindDME function with a Wilcoxon rank‐sum test. Functional enrichment analysis for each module was performed using RunEnrichr with GO Biological Process 2023 and WikiPathways 2024 Mouse, including all genes assigned to the respective module. The enrichment terms were ranked by the combined score, which integrates the significance of gene set overlap (log‐transformed *P* value from a Fisher exact test) with the deviation from expected rank (*z* score) for each term in the gene set library.

#### 2.6.5 Differential gene expression analysis

Differential gene expression analysis per cluster comparing 5XFAD, PS19, and 5XFAD;PS19 to WT was conducted using FindMarkers in Seurat with the Model‐based Analysis of Single‐cell Transcriptomics (MAST, v1.24.1) test. A generalized linear model was applied, incorporating gene detection rate per nucleus as a covariate, and *P* values were adjusted using Bonferroni correction. Genes were considered differentially expressed genes (DEGs) if they met a fold‐change threshold of 3 and a Bonferroni‐adjusted *P* value ≤ 0.05. DEG overlap across genotypes was visualized using ComplexUpset (v1.3.3). Functional enrichment analysis per each cluster and genotype was performed using enrichR (v3.4).^35^ The resulting Gene Ontology (GO) terms were categorized into specific endophenotypes based on the 19 AD biodomains,^36^ using definition files obtained from the Synapse data repository (SynID: syn25428992, syn26856828).

#### 2.6.6 Cell–cell communication analysis with CellChat

Cell–cell communication was inferred using CellChat (v2.1.2)^24^ with default settings. For each genotype, a CellChat object was created from the RNA assay of the Seurat object using the mouse CellChatDB, and the resulting objects were merged for comparative analysis. The total number of inferred interactions and overall interaction strength were compared across genotypes using the compareInteractions function. Inferred interaction is based on the law of mass action using the average expression levels of ligands and receptors between sender and receiver clusters and modeled by a Hill function to account for non‐linear signaling dynamics. Functionally similar signaling pathways across conditions were identified using computeNetSimilarityPairwise, and global communication patterns between cell types and signaling pathways were identified using identifyCommunicationPatterns. The number of patterns was determined by the selectK function in NMF (v0.28). Finally, the overall information flow of each signaling pathway was quantified and ranked using the rankNet function, and the statistical difference between 5XFAD;PS19 and WT was evaluated using a paired Wilcoxon test.

#### 2.6.7 Functional alignment with human AD datasets

For comparison with human bulk RNA‐seq data from the Accelerating Medicines Partnership Program for Alzheimer's Disease (AMP‐AD), we performed differential gene expression analysis on pseudobulked snRNA‐seq data aggregated by sample and genotype. We applied the DESeq2 test to identify DEGs for each genotype (5XFAD, PS19, and 5XFAD;PS19) relative to WT, with significance defined by a Bonferroni‐corrected *P* value < 0.05.

Processed AMP‐AD human bulk RNA‐seq data files, including co‐expression modules (SynID: syn11932957) and log2FC values of the module genes (SynID: syn14237651), were obtained from Synapse. As previously described by Wan et al.,^37^ 30 co‐expression modules were derived from a meta‐analysis of differential gene expression between AD patients and healthy controls across seven distinct brain regions in *post mortem* tissue samples from three large transcriptomic studies.^37^, ^38^, ^39^, ^40^ These modules were further classified into five consensus clusters, each annotated using the Reactome Pathway Database to represent major functional alterations in human AD.^37^, ^41^

Mouse‐human orthologs, previously identified by Wan et al.^37^ using the Comparison of Orthology Predictions (HCOP) tool from HUGO Gene Nomenclature Committee, were obtained from Synapse (SynID: syn17010253). Mouse and human datasets were aligned by gene symbol, and the Pearson correlations between log2FC values from human (AD vs. control) and each mouse model (5XFAD, PS19, or 5XFAD;PS19 vs. WT) were computed using the cor.test function in R.

To identify functionally aligned biological processes between mouse models and human AD, we performed gene set enrichment analysis (GSEA)^42^ for GO terms. DEGs were ranked by average log2FC, and enrichment was tested using fgsea (v1.32.4). GO terms were then categorized into AD biodomains^36^ (SynID: syn25428992, syn26856828). Enrichment scores were normalized for gene set size, returning a normalized enrichment score (NES), in which positive NES values indicate upregulation and negative values indicate downregulation. To assess concordance, NES values for individual enrichment terms were classified as negative (−1), neutral (0), or positive (+1). For each mouse model, the NES direction was then compared to that of the human datasets, with direction signs summed across the three cohorts for each AD biodomain.

## 3 RESULTS

### 3.1 Distinct cell‐population changes in the combined‐pathology mouse model, but not in the Aβ amyloidosis or tauopathy models

To investigate how Aβ pathology, tau pathology, and their combination affect the brain transcriptome, we performed snRNA‐seq on the cortex of WT, 5XFAD^11^ (Aβ amyloidosis), PS19^12^ (tauopathy), and 5XFAD;PS19^43^ (combined pathology) mice (Figure 1A). We used 9‐month‐old animals, when each model exhibits robust amyloid and/or tau pathology,^11^, ^12^, ^43^ and included equal numbers of males and females. Genotype‐specific pathology was validated by quantifying well‐established pathological forms of Aβ and tau levels: 5XFAD exhibited elevated Aβ42, PS19 presented increased phosphorylated tau at threonine 231 (p‐tau‐T231), and 5XFAD;PS19 harbored both pathological features, confirming it as a combined‐pathology model (Figure 1B).

To acquire exceptionally clean snRNA‐seq data, we established a machine‐assisted nuclei isolation protocol that has been shown to yield high‐quality nuclei with extremely low contamination (median mitochondrial reads < 0.5%, median ribosomal reads < 0.03%) across all cell types.^22^ Automation minimized technical variability and ensured greater consistency in key quality control metrics (Figure S1 in supporting information). Furthermore, we applied rigorous preprocessing, including ambient RNA removal using SoupX,^28^ sequencing depth correction via SCTransform,^29^ and sample integration and batch correction using Harmony^30^ (Figure 1C). After this preprocessing pipeline, we retained 170,918 high‐quality nuclei. Quality metrics demonstrated robust data with a median of ≈ 3000 unique genes per nucleus, > 6000 transcripts per nucleus, and < 1% mitochondrial reads (Figure S1A–C). We identified 38 transcriptionally distinct clusters, corresponding to eight major brain cell types: excitatory neurons, inhibitory neurons, oligodendrocytes, microglia, astrocytes, oligodendrocyte precursor cells (OPCs), endothelial cells, and fibroblasts (Figure 1D). Key marker genes for each cell type were highly expressed in their respective clusters (Figure 1E and Table S1 in supporting information).

After annotating each cell type, we examined whether cell proportions varied among the four genotypes (Table S2 in supporting information). The 5XFAD;PS19 model exhibited significant alterations in cell‐type proportions compared to WT, whereas 5XFAD or PS19 alone did not (Figure 1F). This indicates that substantial cell population changes are driven by the combined pathology. Specifically, in the 5XFAD;PS19 model, there was a decrease in excitatory neurons and astrocytes and an increase in oligodendrocytes and microglia (Figure 1F). Further breakdown by cluster for each affected cell type revealed consistent directional trends across most clusters. Notably, ExN_11 (excitatory neurons) and O_3 (oligodendrocytes) exhibited significant changes in 5XFAD;PS19 versus WT (Figure 1G,H). While microglial clusters showed a similar trend of increase in 5XFAD;PS19 mice, this did not reach statistical significance (Figure 1I). All three astrocyte clusters decreased in 5XFAD;PS19 compared to WT (Figure 1J). Taken together, only the combined‐pathology model, unlike the single‐pathology 5XFAD or PS19 models, exhibited distinct cell‐population changes as identified by snRNA‐seq.

### 3.2 Widespread alteration of gene co‐expression networks in the combined‐pathology mouse model

**FIGURE 2:**
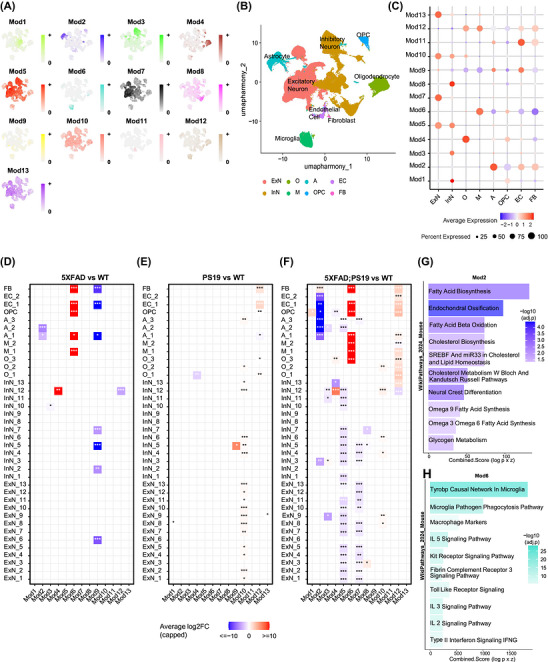
hdWGCNA reveals widespread co‐expression changes in 5XFAD;PS19 mouse. A, Feature plots of hub gene scores for each module (Mod 1–13). The color scale represents hub gene scores based on module eigengene‐based connectivity (kME), indicating pairwise correlations between genes and module eigengenes. B, UMAP showing eight cell types: excitatory neurons (ExN), inhibitory neurons (InN), oligodendrocytes (O), microglia (M), astrocytes (A), oligodendrocyte precursor cells (OPC), endothelial cells (EC), and fibroblasts (FB). C, Dotplot of module expression across cell types. Dot color indicates the module's average scaled expression (blue = low, red = high). Dot size represents the percentage of nuclei within that cell type expressing the module. D–F, Differential module eigengene analysis identifying significant up‐ or downregulation of gene co‐expression in the 13 modules across all clusters comparing 5XFAD versus WT (D), PS19 versus WT (E), and 5XFAD;PS19 versus WT (F), using the Wilcoxon rank‐sum test. Log2FC is visualized with blue (down) to red (up) color scale, with values ranging from –22 to 41, capped at ± 10 to enhance contrast. Bonferroni‐adjusted *P* value is denoted as *P < 0.05, **P < 0.01, ***P < 0.001. Stars are colored white when the absolute fold change is ≥ 3. G–H, Enrichment analysis of Module 2 (G) and Module 6 (H) based on WikiPathways 2024 Mouse. Bar colors correspond to the module colors shown in the feature plot in (B), with the gradient indicating the −log10 of the Benjamini–Hochberg adjusted *P* value. The combined score integrates the significance of gene set overlap (log p) with the deviation from expected rank (*z* score) for each term in the gene set library. hdWGCNA, high‐dimensional weighted gene co‐expression network analysis; UMAP, uniform manifold approximation and projection; WT, wild type

To investigate global patterns of gene expression first, we applied hdWGCNA^23^ across all clusters. We identified 13 co‐expression modules, each representing a group of genes with highly correlated expression and localized to specific cell types (Figure 2A–B and Table S3 in supporting information). Quantifying module expression across clusters revealed cell type–specific enrichment; for example, Mod4 was expressed predominantly in oligodendrocytes (Figures 2C and S2A in supporting information).

To assess genotype‐dependent perturbations of co‐expression networks, we performed differential module eigengene analysis (Table S3). The 5XFAD;PS19 model exhibited the most pronounced module dysregulation compared to WT, far exceeding the changes observed in either 5XFAD or PS19 alone (Figure 2D–F). In 5XFAD mice, only a limited number of modules were significantly altered. Mod2 was downregulated in a subset of astrocyte clusters (A_1 and A_2), and Mod9 was reduced in several neuronal and glial clusters. Conversely, Mod6 was upregulated in glial populations including microglia (M_1) and astrocytes (A_1; Figure 2D). In PS19 mice, module‐level changes were comparatively sparse. Mod4 and Mod9 alterations were each restricted to a single cluster, whereas Mod10 and Mod12 exhibited broader but modest shifts with small fold changes (Figure 2E). In contrast, the combined‐pathology model exhibited widespread, high‐magnitude module dysregulation across numerous clusters (Figure 2F). Several modules that changed in 5XFAD or PS19 alone were not only recapitulated but markedly expanded in 5XFAD;PS19. For example, Mod2 downregulation presents only in A_1 and A_2 in 5XFAD (Figure 2D), extended to all astrocyte clusters (A_1, A_2, and A_3), OPCs, endothelial cells (EC_1 and EC_2), and inhibitory neurons (InN_3) in the combined model, with substantially larger fold changes (Figure 2F). Mod6 upregulation also intensified, with an additional increase in microglial (M_2) and oligodendrocyte (O_3) clusters uniquely in 5XFAD;PS19 (Figure 2F). Similarly, Mod12 showed only modest changes in PS19 (Figure 2E) but became strongly upregulated across many clusters in the combined model (Figure 2F). Notably, Mod5 and Mod7, expressed predominantly in neuronal populations, were modestly downregulated only in 5XFAD;PS19 (Figure 2F).

To identify biological pathways represented by each module, we performed functional enrichment analysis using the enrichR^35^ R package. Mod2 was enriched for lipid metabolic processes, including fatty acid and cholesterol biosynthesis (Figure 2G), suggesting dysregulation of lipid metabolism in 5XFAD;PS19. Mod6 was enriched for microglial activation, immune signaling, and phagocytic pathways (Figure 2H), reflecting the strong alteration in immune systems in both 5XFAD and 5XFAD;PS19. Although other modules were not significantly enriched in Wikipathways, GO analysis revealed that Mod5 and Mod7 were associated with glutamatergic and GABAergic synaptic transmission, respectively (Figure S2B,C). These functional annotations support the downregulation of excitatory and inhibitory synaptic programs particularly in the combined pathology. Together, these findings highlight substantial alterations in functional gene co‐expression networks in both glia and neurons of the combined pathology model, with glial modules exhibiting particularly pronounced differential regulation.

### 3.3 Exclusive and synergistic transcriptional changes in the combined‐pathology mouse model

**FIGURE 3:**
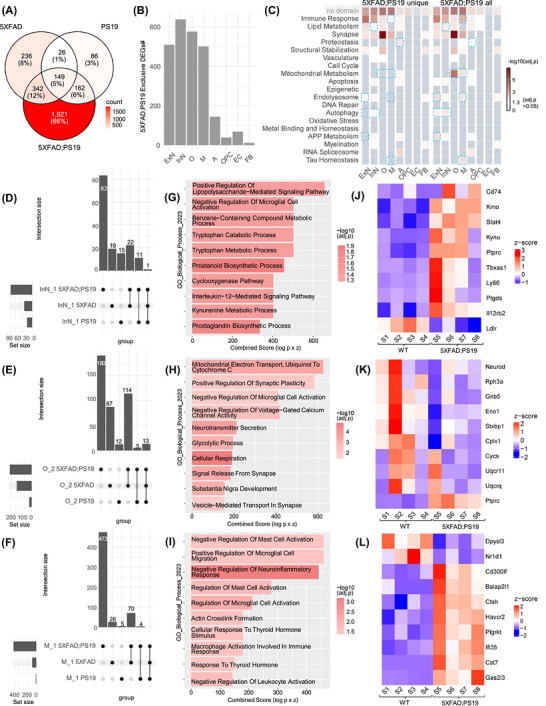
Differential gene expression analysis reveals extensive transcriptional changes specific to the 5XFAD;PS19 model. A, Venn diagram of DEGs detected in 5XFAD, PS19, and 5XFAD;PS19 versus WT. Sections are color‐coded from white (fewer DEGs) to red (more DEGs). Results were filtered by an absolute fold change ≥ 3 and an adjusted *P* value (Bonferroni) ≤ 0.05. B, Bar graph showing the distribution of DEGs exclusive to 5XFAD;PS19 across each cell type. C, Heatmap of over‐represented enrichment terms for DEGs unique to 5XFAD;PS19 (“5XFAD;PS19 unique,” left) and for all DEGs detected in 5XFAD;PS19 (“5XFAD;PS19 all,” right), stratified by cell type. Terms are grouped by AD biodomains; overly broad terms were labeled as “no domain.” Color indicates ‐log10(adjusted *P* value), from gray (non‐significant) to white (adjusted *P* value = 0.05) to red (highly significant). Dashed boxes in blue indicate notable differences in the statistical significance of enrichments between unique and all DEGs. D–F, UpSet plots showing the number of DEGs (set size) in each group versus WT and their overlap across comparisons for representative clusters of inhibitory neurons (InN_1; D), oligodendrocytes (O_2; E), and microglia (M_1; F). G–I, Enrichment analysis of all 5XFAD;PS19 DEGs in InN_1 (G), O_2 (H), and M_1 (I) filtered for significant terms (–log10 adjusted *P* value > 1.3). The bar color reflects –log10 of Benjamini–Hochberg adjusted *P* value. The combined score integrates the significance of gene set overlap (log p) with the deviation from expected rank (*z* score) for each term in the gene set library. J–L, Heatmaps showing per‐gene *z* scores of the top upregulated or downregulated DEGs driving the enrichment terms in individual WT control samples (S1–S4) versus 5XFAD;PS19 samples (S5–S8) for InN_1 (J), O_2 (K), and M_1 (L). *Z* scores are color‐coded from blue (below mean), through white (mean), to red (above mean). AD, Alzheimer's disease; DEG, differentially expressed gene; WT, wild type

To investigate cell type–specific transcriptomic alterations driven by combined amyloid and tau pathologies, we performed differential gene expression analysis across all clusters, comparing each genotype, 5XFAD, PS19, and 5XFAD;PS19, to WT controls. We identified a total of 2922 DEGs in all three comparison groups (Table S4 in supporting information). Remarkably, 1921 of these DEGs (66%) were exclusive to the 5XFAD;PS19 model, suggesting a synergistic effect of the combined pathology not observed in either 5XFAD or PS19 alone (Figure 3A). These unique DEGs spanned multiple cell types (Figure 3B). Excitatory and inhibitory neurons exhibited the greatest number of DEGs, likely reflecting their greater representation, which confers greater statistical power to detect DEGs. In comparison, glial populations, especially oligodendrocytes and microglia, exhibit disproportionately high DEG counts relative to their nucleus abundance (Figures 3B and S3A in supporting information). This finding underscores a substantial glial contribution to the synergistic transcriptional alterations observed in the 5XFAD;PS19 model.

To determine which biological processes were represented by these DEGs, we performed functional enrichment analysis using enrichR.^35^ To interpret these results within a disease‐relevant framework, enriched pathways were organized using AD‐relevant molecular endophenotypes (termed “AD biological domains” or “AD biodomains”).^36^ This biodomain framework was developed by the Emory–Sage–Structural Genomics Consortium–Jackson Laboratory (ESSJ) Target Enablement to Accelerate Therapy Development for AD (TREAT‐AD) center to objectively categorize genes into 19 AD‐associated endophenotypes based on GO terms.^36^ The framework has been applied to compare pathway‐level effects in both transcriptomic^44^ and proteomic datasets.^45^ Using this approach, we found that the DEGs unique to 5XFAD;PS19 were strongly enriched in immune response, lipid metabolism, and synaptic function (Figure 3C, left; Table S4). When the analysis was expanded to include all 5XFAD;PS19 DEGs, including those overlapping with 5XFAD and/or PS19, additional enriched AD biodomains emerged, such as mitochondrial metabolism, endolysosomal processing, APP metabolism, and tau‐related pathways (Figure 3C, right). Interestingly, microglial enrichment terms involved in immune response, mitochondrial metabolism, endolysosomal function, and tau homeostasis reached significance only when all DEGs in 5XFAD;PS19 mice were included (Figure 3C, right). This pattern suggests that alterations in these microglial functions are largely shared across the single and combined pathologies.

To further dissect cell type–specific alterations, we examined representative clusters, typically the largest, for each major cell type. We demonstrated that 5XFAD;PS19‐unique DEGs constituted the largest fraction of DEGs in every cell type, as shown in the representative clusters of inhibitory neurons (InN_1; Figure 3D), oligodendrocytes (O_1, O_2; Figure S3B,E), microglia (M_1; Figure 3F), excitatory neurons (ExN_1; Figure S3C), astrocytes (A_1; Figure S3D), endothelial cells (EC_1; Figure S3E), and fibroblasts (FB; Figure S3F). Enrichment analysis of all DEGs in 5XFAD;PS19 mice revealed immune signaling in InN_1 (Figure 3G), mitochondrial and synaptic function in O_2 (Figure 3H), and immune‐related functions in M_1 (Figure 3I).

Other representative clusters presented relatively few significant enrichments (Figure S3G–K). To assess the effect size of these changes, we visualized the expression levels of DEGs contributing to enrichment terms across the WT and 5XFAD;PS19 samples in each representative cluster (Figure 3J–L). In the InN_1 cluster, immune‐related genes such as *Kmo* and *Stat4* were upregulated, whereas *Ldlr* was downregulated in 5XFAD;PS19 compared to WT (Figure 3J). In the O_2 cluster, the immune gene *Ptprc* (Cd45) was elevated, whereas synaptic genes including *Rph3a* and *Stxbp1* were reduced (Figure 3K). In the M_1 cluster, the actin‐associated gene *Dpysl3* was downregulated, whereas the lysosomal genes *Ctsh* and *Cst7* were upregulated in 5XFAD;PS19 relative to WT (Figure 3L). These heatmaps captured overall differences between WT and 5XFAD;PS19, revealing robust transcriptional shifts in the combined pathology model. Together, differential gene expression analyses demonstrated that combined pathology induces exclusive transcriptional alterations, not observed in single‐pathology mouse models (amyloid only or tau only), with substantial contributions from inhibitory neurons, oligodendrocytes, and microglia.

### 3.4 Microglia and oligodendrocyte clusters enriched in the combined pathology mouse model exhibit synapse‐associated transcriptional suppression

**FIGURE 4:**
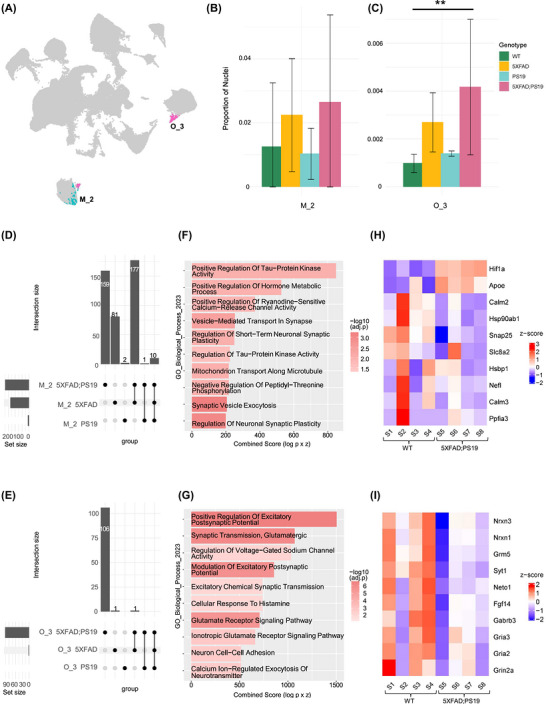
Microglia and oligodendrocyte clusters in 5XFAD;PS19 mice share synapse‐related transcriptional suppression. A, UMAP plot highlighting microglia cluster M_2 (blue) and oligodendrocyte cluster O_3 (pink). B–C, Nuclei proportions across genotypes in M_2 (B) and O_3 (C). Error bars represent standard deviation (*P < 0.05, **P < 0.01). D–E, UpSet plots showing the number of differentially expressed genes (set size) in each group versus wild type and their overlap across comparisons for clusters of M_2 (D) and O_3 (E). F–G, Enrichment analysis for M_2 (F) and O_3 (G), filtered for significant terms (–log10 adjusted *P* value > 1.3). The bar color reflects –log10 of Benjamini–Hochberg adjusted *P* value. The combined score integrates the significance of gene set overlap (log p) with the deviation from expected rank (*z* score) for each term in the gene set library. H–I, Heatmaps showing per‐gene *z* scores of the top five upregulated and bottom five downregulated DEGs contributing to enriched terms in the individual samples of WT (S1–S4) versus 5XFAD;PS19 (S5‐S8) in M_2 (H) and O_3 (I). *Z* scores are color‐coded from blue (below mean), through white (mean), to red (above mean). DEG, differentially expressed gene; UMAP, uniform manifold approximation and projection; WT, wild type

To further explore transcriptomic alterations in glial populations, we focused on M_2 microglia and O_3 oligodendrocytes, two clusters overrepresented in the 5XFAD;PS19 model compared to WT. Cluster O_3, in particular, represented the largest proportion in the combined pathology model compared to the other groups (Figure 4A–C). Notably, the expression of hdWGCNA Mod6 was enriched in both M_2 and O_3, with especially strong upregulation in 5XFAD;PS19 (Figure 2F), suggesting that these clusters participate in a shared glial co‐expression network responsive to combined amyloid and tau pathology. Moreover, the O_3 cluster was distinctly enriched for marker genes of disease‐associated oligodendrocytes (DAO)^17^, ^46^ compared to other oligodendrocyte clusters, with the DAO signature most prominently expressed in the 5XFAD;PS19 model (Figure S4A,B in supporting information). In the M_2 cluster, the 5XFAD;PS19 model also exhibited enrichment of several marker genes associated with specific microglial states, including disease‐associated microglia (DAM)‐like, antigen presentation (HLA/MHC class II), and cytokine response microglia involved in inflammation (Figure S4C–E).^47^

Differential gene expression analysis revealed a substantial number of DEGs unique to 5XFAD;PS19 in both M_2 microglial and O_3 oligodendrocyte clusters (Figure 4D,E). Notably, in the M_2 cluster, 5XFAD;PS19 shared 177 DEGs with 5XFAD but only 1 with PS19 (Figure 4D). Enrichment analysis of these DEGs uncovered several overrepresented terms related to synaptic function, including “vesicle‐mediated transport in synapse” and “synaptic vesicle exocytosis” in M_2 (Figure 4F) and “regulation of excitatory postsynaptic potential” and “synaptic transmission, glutamatergic” in O_3 (Figure 4G).

Notably, the DEGs contributing to these synapse‐related enrichments were downregulated in 5XFAD;PS19 compared to WT in both M_2 and O_3 (Figure 4H,I). In M_2, genes involved in calcium signaling and synaptic vesicle function (e.g., *Calm1, Calm2, Calm3, Snap25*, and *Vamp2*) were suppressed. In contrast, *Hif1a*, a transcription factor induced under metabolic stress or hypoxia, and apolipoprotein E, encoding a lipid transporter, were uniquely upregulated, potentially reflecting a compensatory or stress‐responsive mechanism (Figures 4H and S4F). In O_3, genes involved in synaptic transmission and neuron–glia interactions (e.g., *Nrxn1, Nrxn3, Neto1, Gria1, Gria2, Gria3*, and *Cntn4*) were downregulated (Figure 4I,G). Although synaptic functions are classically neurocentric, such terms in glial clusters likely reflect neuron–glia communication. These findings suggest that microglia and oligodendrocytes may be engaged in transcriptionally coordinated responses linked to reduced synaptic regulation and neuron–glia communication in the context of combined amyloid and tau pathology.

### 3.5 Enhanced cell–cell communication in the combined‐pathology model, partially driven by microglial and oligodendrocyte clusters through immune pathways

**FIGURE 5:**
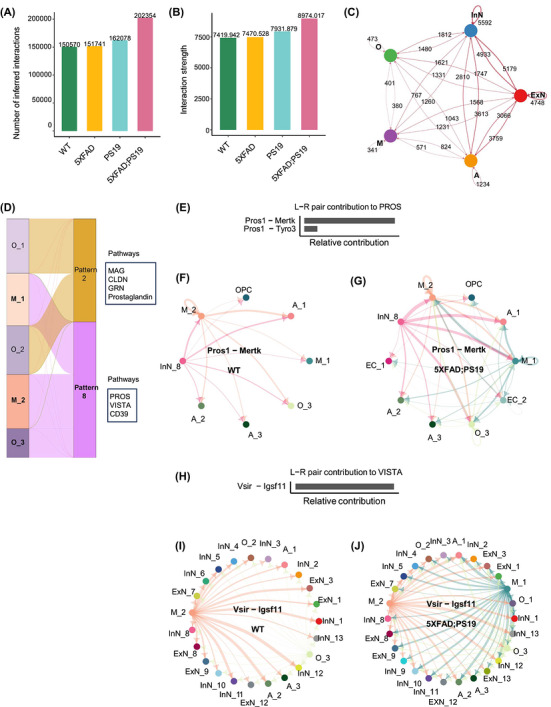
Enhanced cell–cell communication in the 5XFAD;PS19 model, partially driven by microglial and oligodendrocyte clusters. A–B, Bar plots of the total number of inferred interactions (A) and overall communication strength (B) across genotypes, using the average expression levels of ligands and receptors between sender and receiver clusters. C, Circle plot of the differential number of interactions among major cell types in 5XFAD;PS19 compared to WT. Each node represents a cell type, and each edge represents directional interaction. Edge width represents the absolute number of differentially increased interactions. D, Alluvial plot illustrating outgoing communication patterns in 5XFAD;PS19, with corresponding clusters (left) and signaling pathways (right) in coordination. The thickness of the flow indicates the contribution of the cluster to the corresponding communication pattern. E–G, PROS signaling. E, Relative contribution of each ligand–receptor pair to the overall PROS pathway. F–G, Circle plots of Pros1–Mertk ligand–receptor pair in PROS signaling pathway in WT (F) and 5XFAD;PS19 (G). H–J, VISTA signaling. H, Relative contribution of a ligand‐receptor pair to the overall VISTA pathway. I–J, Circle plots of Vsir–Igsf11 ligand–receptor pair in VISTA signaling pathway in WT (I) and 5XFAD;PS19 (J). In all circle plots, each node represents a cluster, and each directed edge indicates a signaling interaction. Edge color corresponds to the sender cluster, and edge thickness reflects the interaction strength, with thicker lines indicating stronger signals. PROS, protein S; VISTA, V‐set immunoregulatory receptor; WT, wild type

To investigate how combined amyloid and tau pathology impacts intercellular communication, we applied CellChat^24^ to infer ligand–receptor interactions across genotypes (Table S5 in supporting information). The inferred total number of interactions and overall signaling strength were greater in 5XFAD;PS19 than in single‐pathology models and WT controls (Figure 5A,B). While the largest number of interactions involved excitatory and inhibitory neurons, likely reflecting their greater representation, glial populations such as microglia, oligodendrocytes, and astrocytes also presented increased signaling activity (Figure 5C).

To examine how different cell clusters and signaling pathways coordinate communication, we performed CellChat's pattern recognition analysis for outgoing and incoming communications in 5XFAD;PS19 (Figure S5A,B in supporting information). Among the nine outgoing communication patterns identified, Pattern 8 was driven predominantly by microglial clusters M_1 and M_2 and oligodendrocyte cluster O_3, the clusters that co‐express hdWGCNA Mod6 (Figure S2A). Pattern 8 was associated primarily with three immune‐related signaling pathways: PROS (Protein S), VISTA (a receptor also known as VSIR, V‐set immunoregulatory receptor), and CD39 (Figure 5D). Pathway‐level information flow analysis predicted that PROS and VISTA signaling were significantly elevated in 5XFAD;PS19 compared to WT, as determined via a paired Wilcoxon test (Figure S5C).

The inferred changes in PROS pathway were mainly mediated by PROS1–MERTK, with a smaller contribution from PROS1–TYRO3 ligand–receptor pairs (Figure 5E). PROS1 acts as an anti‐inflammatory mediator by activating TAM (TYRO3, AXL, and MERTK) receptors, facilitating the phagocytic clearance of apoptotic cells and dampening inflammation.^48^ In WT controls, PROS signaling was driven mostly by InN_8 and M_2, whereas in 5XFAD;PS19, outgoing signals increased from InN_8, O_3, M_1, M_2, and EC_2 (Figures 5F,G, and S6A in supporting information). Incoming communication also intensified in microglia (M_1–M_2) and astrocytes (A_1–A_3), as captured by the incoming communication Pattern 2 (Figure S5B).

The predicted changes in VISTA pathway, mediated by the single receptor‐ligand pair VSIR–IGSF11 (immunoglobulin superfamily member 11; Figure 5H), also showed enhanced signaling in 5XFAD;PS19. Outgoing signaling increased from M_1 and O_3 clusters, with broader reception across both neuronal and glial clusters (Figures 5I,J, and S6B). VISTA functions as an inhibitory immune checkpoint, attenuating T‐cell activation and contributing to immune homeostasis.^49^, ^50^

Together, these cell–cell communication inferences suggest that the 5XFAD;PS19 model enhances intercellular communication across brain cell types. This increase was partially driven by microglial and oligodendrocyte clusters, which seem to respond to combined pathology by upregulating immune‐modulatory signaling, particularly via anti‐inflammatory and immunosuppressive pathways.

### 3.6 The 5XFAD;PS19 model recapitulates key transcriptomic features of human AD

**FIGURE 6:**
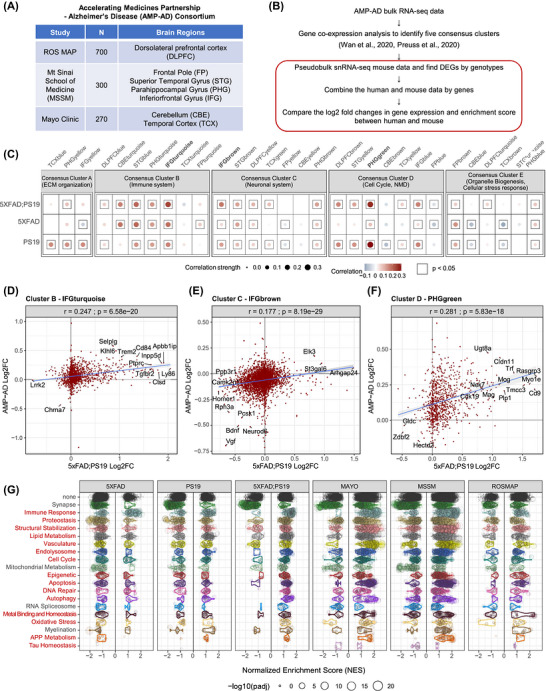
Cross‐species comparison reveals significant correlations between 5XFAD;PS19 and human AD transcriptomes. A, Overview of AMP‐AD human *post mortem* RNA‐seq data from ROSMAP, MSSM, and Mayo Clinic studies. B, Schematic of pipelines to align mouse and human gene expression; steps in the red box were performed in this study to compare DEGs between human AD and mouse models. C, Pearson correlation between gene expression changes in transgenic mouse models (rows) and human AD patients, across 30 AMP‐AD co‐expression modules grouped into five consensus clusters (columns). Positive correlations are shown in red and negative correlations in blue. Circle size is proportional to the correlation strength. Black squares denote significant correlations (*P* < 0.05). D–F, Pearson correlations between log2FC of DEGs in AMP‐AD human data and the 5XFAD;PS19 mouse model for the IFGturquoise module in Cluster B (D), IFGbrown in Cluster C (E), and PHGgreen in Cluster D (F). The blue line indicates the linear regression fit, and shaded gray area represents the 95% confidence interval. Genes with large absolute fold changes along the regression line, as well as genes with strong concordant changes between mouse and human, are labeled. G, NES across mouse models and AMP‐AD human studies. GO terms identified by GSEA are grouped into AD biodomains (rows). Biodomains in which 5XFAD;PS19 exhibit the greatest number of concordant enrichment terms with human AD (relative to 5XFAD or PS19 alone) are highlighted in red. Dot size reflects –log10(adjusted *P* value) for each GO term. AD, Alzheimer's disease; AMP‐AD, Accelerating Medicines Partnership Program for Alzheimer's Disease; DEG, differentially expressed gene; ECM, extracellular matrix; GO, Gene Ontology; GSEA, gene set enrichment analyses; MSSM, Mt. Sinai School of Medicine; NES, normalized enrichment score; NMD, nonsense‐mediated messenger RNA decay; ROSMAP, Religious Orders Study and Memory and Aging Project

To assess the translational relevance of our mouse models, we conducted a cross‐species comparison with transcriptomic data from the AMP‐AD consortium. This resource includes *post mortem* RNA‐seq datasets from the Religious Orders Study and Memory and Aging Project (ROSMAP)^40^, Mount Sinai School of Medicine (MSSM),^39^ and Mayo Clinic,^38^ encompassing > 1200 individuals across seven brain regions (Figure 6A).

We leveraged a previously published analysis of the AMP‐AD data, which identified 30 co‐expression modules significantly enriched for DEGs between AD patients and controls.^37^ These modules were grouped into five consensus clusters and annotated using Reactome, representing conserved AD‐related molecular signatures across cohorts and brain regions.^37^, ^41^, ^51^ For comparison, we generated pseudobulk transcriptomes from our snRNA‐seq data by aggregating gene expression across nuclei by sample. DEGs were calculated for 5XFAD, PS19, and 5XFAD;PS19 versus WT using a fold‐change threshold of 3 and a Bonferroni‐corrected *P* value ≤ 0.05. Mouse genes were then mapped to human orthologs, and log2FC and enrichment scores were compared between mice and humans (Figure 6B).

Correlation analysis revealed that DEGs from the 5XFAD;PS19 model exhibited the strongest significant alignment with DEGs from human AD transcriptomes (Table S6 in supporting information), particularly in modules related to immune response (Cluster B), neuronal signaling (Cluster C), mitotic cell cycle or nonsense‐mediated mRNA decay pathways (Cluster D), and organelle biogenesis or cellular stress response pathways (Cluster E; Figure 6C). 5XFAD and PS19 each alone generally presented weaker or non‐significant correlations in those clusters (Figure 6C). Gene‐level correlation plots for representative modules identified genes with functions linked to each module (Table S6). IFGturquoise (Cluster B) represented the upregulation of immune‐related genes such as *Inpp5d* (regulation of immune cell signaling), *Selplg* (leukocyte trafficking), and *Cd84* (signaling lymphocyte activation molecules; Figure 6D). IFGbrown (Cluster C) represented the downregulation of synapse‐related genes, including *Vgf* and *Bdnf* (regulation of synaptic activity and plasticity; Figure 6E), and PHGgreen (Cluster D) represented the upregulation of cell cycle–related genes, such as *Nek7* (Figure 6F). Notably, PHGgreen was enriched with oligodendrocyte‐ and myelin‐associated genes (*Mog, Mag, Cldn11*, and *Ugt8a*; Figure 6F), consistent with findings from Wan et al.^37^

We further assessed pathway‐level alignment via GSEA,^42^ comparing NES across our mouse models and human AMP‐AD datasets from three cohorts (Figure 6G) representing seven brain regions (Figure S7A and Table S7 in supporting information). Enriched pathways were then mapped to the 19 AD biodomains to help bridge gene‐specific differences between human and mouse models by focusing on phenotypes we can observe in both species. The 5XFAD;PS19 model exhibited the greatest number of enrichment terms with NES values concordant with the human datasets in 15 out of the 19 AD biodomains, including immune response, lipid metabolism, and cell cycle (Figure S7B). Together, these results indicate that the 5XFAD;PS19 model recapitulates key molecular features of human AD with higher fidelity than either 5XFAD or PS19 alone does, underscoring its value for investigating AD mechanisms and identifying therapeutic targets relevant to human disease.

## 4 DISCUSSION

This resource study reveals two distinct transcriptional programs that emerge prominently when amyloid and tau pathologies coexist: disruption of glial lipid metabolic networks and coordinated microglial–oligodendrocyte responses involving immune and synaptic alterations. Using machine‐isolated and rigorously preprocessed high‐quality snRNA‐seq data from WT, 5XFAD (Aβ), PS19 (tau), and 5XFAD;PS19, we uncovered multi‐scale transcriptional alterations, encompassing changes in gene co‐expression networks and individual gene expression patterns across major brain cell types. Notably, the combined pathology featured widespread downregulation of a lipid metabolism‐related gene module and upregulation of an immune‐related gene module (Figure 2F–H), alongside suppressed synaptic transmission (Figure 4F–I) and coordinated immune responses (Figure 5D–J) involving both microglia and oligodendrocytes. Our comprehensive and sex‐balanced study dissects the synergistic effects of combined pathology across multiple brain cell types, at both the network and individual gene expression levels, incorporating intercellular interactions. We further compared our mouse data with AMP‐AD human datasets and identified the strongest concordance between 5XFAD;PS19 and human AD across most functional pathways, including immune response, lipid metabolism, and cell cycle reentry (Figure 6G).

hdWGCNA revealed coordinated alterations of glial and neuronal modules in 5XFAD;PS19. The lipid metabolism module (Mod2) was moderately downregulated in some astrocyte clusters of 5XFAD, but not in PS19. Meanwhile, Mod2 was substantially downregulated across all astrocyte clusters, OPC, and endothelial cells in the combined pathology model (Figure 2D–F). This finding aligns with reports that astrocytic lipid metabolism pathways are downregulated in the APP/PS1 amyloidosis model.^52^ It further corresponds with altered lipid metabolites and relevant gene expression in human AD.^53^, ^54^, ^55^ In contrast, the immune module (Mod6) was upregulated in both 5XFAD and 5XFAD;PS19 but not in PS19 (Figure 2D–F), suggesting that Aβ is the primary driver of neuroinflammation. This aligns with human data linking plaque burden to elevated expression of microglial activation markers such as *TREM2*, *TYROBP*, and *CD68*,^56^ as well as exacerbated microgliosis and astrogliosis in 5XFAD.^25^, ^57^ The localization of pro‐inflammatory DAM and DAO to Aβ plaques^16^, ^17^, ^46^ further reinforces the role of Aβ as the primary trigger for these immune responses. Neuronal modules associated with synaptic transmission and calcium signaling (Mod5 and Mod7) were selectively downregulated in 5XFAD;PS19 (Figure 2D–F). Synaptic dysfunction is a hallmark of AD and has been reported in both combined pathology mouse models and human AD brains.^43^, ^58^, ^59^ The alteration of these synaptic modules may reflect the reduction in excitatory neurons in 5XFAD;PS19 (Figure 1F), consistent with the findings from human AD datasets.^60^ Collectively, these module‐level alterations indicate that the Aβ and tau combined model captures lipid metabolic, immune, and synaptic features not represented in single‐pathology models alone.

Differential gene expression analysis further reaffirmed the synergistic effects of combined pathology. A substantial number of DEGs unique to 5XFAD;PS19 were found across all cell types, particularly enriched for immune response, lipid metabolism, and synaptic pathways (Figure 3A–C). The most notable changes were observed in the oligodendrocyte (O_3) and microglia (M_2) clusters, mirroring the network‐level disruptions. In the hdWGCNA analysis, the immune‐related Mod6 was upregulated in both 5XFAD and 5XFAD;PS19. However, only 5XFAD;PS19 showed this upregulation within O_3 and M_2 (Figure 2F), indicating a synergistic effect of combined pathologies in these specific clusters. In the DEG analysis, the O_3 cluster was enriched for the DAO signature, the most overrepresented in the combined model (Figure S4A,B). This aligns with emerging evidence that oligodendrocytes actively respond to AD pathology, transitioning into reactive, “disease‐associated” states marked by immune and stress‐response genes.^61^ Oligodendrocytes are also known to crosstalk with microglia, expressing innate immune receptors and modulating central nervous system (CNS) immunity through cytokine signaling.^62^ Like DAM, which appears in 5XFAD at 1 to 2 months of age, DAO is also enriched near Aβ plaques at 9 to 10 months of age.^17^, ^46^ Interestingly, both M_2 and O_3 exhibited the suppression of synaptic gene expression (Figure 4H,I), with enrichment terms related to synaptic transmission (Figure 4F,G). Oligodendrocytes may modulate axon myelination in response to synaptic input, given their expression of glutamate receptor genes in mice^63^ and of excitatory synaptic proteins in zebrafish.^64^ A snRNA‐seq study in human AD also reported downregulation of synaptic cell adhesion–related gene expression and enrichment terms in an oligodendrocyte cluster.^65^, ^66^

Furthermore, CellChat analysis inferred significantly increased communication among the O_3 and M_2 clusters, with additional involvement of the M_1 cluster, in immune‐relevant pathways such as PROS and VISTA (Figures 5D and S5C). In the PROS pathway, PROS1 acts as a ligand for MERTK, promoting phagocytosis and enhancing astrocyte‐mediated clearance of apoptotic cells in a CNS demyelination model.^67^ In our data, the combined pathology increased outgoing signals from M_1, M_2, and O_3 and elevated incoming signals to astrocyte clusters (Figures 5G and S6A). This suggests that these clusters may engage astrocytes via MERTK to facilitate debris clearance. The VISTA pathway, a known suppressor of T‐cell activation, also exhibited increased signaling from M_1 and O_3 in 5XFAD;PS19 (Figures 5J and S6B). VISTA has been reported to be upregulated in microglia surrounding Aβ plaques in AD but downregulated in chronically inflamed lesions in multiple sclerosis,^49^ suggesting that its regulation may be disease‐ and context‐dependent. As VISTA remains understudied in AD, our findings highlight the need to investigate whether its upregulation serves as a compensatory mechanism to dampen neuroinflammation.

Importantly, pathway‐level transcriptomic changes in our combined model align closely with human AD. Prior cross‐species analyses found that 5XFAD showed the greatest overlap with human AD in terms of immune activation and synaptic downregulation.^68^ In our analysis, 5XFAD;PS19 demonstrated a correlation with human data comparable to 5XFAD in the immune system but exhibited stronger alignment in extracellular matrix organization, neuronal system (synapse), and cell cycle consensus clusters (Figure 6C). Moreover, the combined model outperformed 5XFAD and PS19 with a greater number of enrichment terms concordant with human data across most biodomains, including immune response, lipid metabolism, and cell cycle (Figure S7B). These findings highlight the synergistic effects in 5XFAD;PS19, particularly within immune and lipid metabolism pathways, as supported by both gene network and DEG analyses (Figures 2F and 3C). Additionally, a prior study reported a 3‐ to 4‐fold increase in the number of neurons expressing cell cycle reentry programs in advanced AD patients compared to healthy individuals.^69^ These results suggest that combined pathology is especially useful for capturing multicellular glial and synaptic programs that are highly relevant to human AD biology.

Several limitations should be considered. First, although 5XFAD;PS19 provides an experimentally tractable system for studying Aβ–tau interactions, parental lines rely on transgenic overexpression of familial AD or frontotemporal dementia (FTD) mutations and do not fully recapitulate the slow accumulation of amyloid and tau in humans.^70^, ^71^ Nevertheless, proteomics comparison demonstrated that amyloid mouse models, including 5XFAD, capture ≈ 30% of human AD‐associated protein alterations.^72^ More physiological double knock‐in models express humanized mutant *APP* and *MAPT* under endogenous promoters.^73^, ^74^ However, APP^NL‐G‐F^/MAPT do not develop neurofibrillary tangles and neuronal loss until 24 months,^73^ and remain transcriptomically undercharacterized. It will be informative to compare the transcriptional programs identified here with future snRNA‐seq studies with such knock‐in models. Second, CellChat inferences represent computationally derived communication probabilities from gene expression data and a curated ligand‐receptor database. CellChat does not capture the full complexity of in vivo signaling dynamics, including post‐translational regulation and spatial constraints. Therefore, the predicted signaling should be interpreted as hypothesis‐generating and requires experimental validation in future studies. Third, the cross‐species comparison is constrained by differences in tissue context, disease staging, and analytical framework. Our mouse data derive from snRNA‐seq of acutely isolated cortex analyzed using pseudobulk aggregation. Conversely, AMP‐AD data consist of human *postmortem* bulk RNA‐seq across multiple brain regions reflecting heterogeneous disease stages, comorbidities, and technical factors. Particularly, *post mortem* interval (PMI) warrants careful interpretation. A 3‐hour PMI in PS19 at 4°C before tissue processing substantially reduces the disease‐associated transcriptional signature in neurons, despite minimal effects on RNA Integrity Number (RIN).^75^ Although these considerations do not invalidate human brain transcriptomics or cross‐species comparisons, they warrant cautious interpretation of concordance metrics and highlight the value of well‐controlled mouse datasets as complementary resources for resolving disease‐associated transcriptional programs in brain diseases. To mitigate gene‐level and species‐specific differences, we focused on conserved, disease‐relevant pathway‐ and biodomain‐level concordance. Therefore, human concordance results should be interpreted as evidence of pathway‐level similarity.

These findings have important practical implications for the interpretation and design of AD preclinical studies. Modeling Aβ and tau separately versus together may yield different conclusions about lipid dysregulation, glial coordination, and immune network remodeling. Our dataset therefore provides a biological reference for distinguishing transcriptomic programs that are broadly shared from those most apparent when both pathologies coexist. More broadly, this resource offers a foundation for systems‐level therapeutic discovery in AD. It resolves cell type–specific network changes, shared versus pathology‐specific responses, and candidate intercellular signaling pathways, enabling network‐based target prioritization, identification of multicellular intervention points, and integration with human genetic and pharmacologic evidence. To further support the research community, our snRNA‐seq dataset is publicly available and can be queried to examine cell type–specific transcriptomic changes attributable to Aβ, tau, or their interaction. Researchers can leverage this resource to investigate pathological mechanisms relevant to specific biological questions, and generate new hypotheses grounded in multi‐pathology transcriptomics.

## AUTHOR CONTRIBUTIONS

Jung Hyun Park, Luke Child Dabin, Byungwook Kim, and Jungsu Kim conceived and designed the project. Jung Hyun Park, Luke Child Dabin, Byungwook Kim, Mason Douglas Tate, Ahmad Daniel Sharify, and Sutha K. John performed the experiments. Jung Hyun Park, Luke Child Dabin, and Byungwook Kim analyzed the data. Jung Hyun Park drafted the manuscript. All authors revised and approved the final manuscript.

## CONFLICT OF INTEREST STATEMENT

The authors declare no competing interests. Author disclosures are available in the Supporting Information.

## Supporting information




**Supporting Information**: supp‐info‐0001‐figures.docx

**Supporting Information**: supp‐info‐0002‐tableS1.xlsx

**Supporting Information**: supp‐info‐0003‐tableS2.xlsx

**Supporting Information**: supp‐info‐0004‐tableS3.xlsx

**Supporting Information**: supp‐info‐0005‐tableS4.xlsx

**Supporting Information**: supp‐info‐0006‐tableS5.xlsx

**Supporting Information**: supp‐info‐0007‐tableS6.xlsx

**Supporting Information**: supp‐info‐0008‐tableS7.xlsx

**Supporting Information**: alz71742‐sup‐0009‐SuppMat.pdf

## Data Availability

The definitions and labels of AD biodomains are accessible at the Synapse data repository (SynID: syn25428992, syn26856828). The AMP‐AD human bulk RNA‐seq data files are accessible at Synapse (SynID: syn11932957, syn14237651). Mouse‐human orthologs for the AMP‐AD human bulk RNA‐seq data are accessible at Synapse (SynID: syn17010253). Materials generated during this study are accessible via reasonable request to the corresponding authors’ labs. Our snRNA‐seq data were uploaded to GEO Accession **GSE304499** (reviewer token: atgdyiuohjktfyh). Code for transcriptomic data analysis and cross‐species alignment using standard packages as described in the STAR Methods is available on GitHub at https://github.com/jungsukimlab/.
