# Supplementary material for: Unique transcriptomic alterations in 5XFAD;PS19 mouse model identify glial lipid dysregulation and coordinated microglial–oligodendrocyte responses
Source: Alzheimers Dement. 2026 Aug 10;22(8):e71742. doi: 10.1002/alz.71742 (PMC13457352; doi:10.1002/alz.71742)
Supplement: Supplementary file 2 [file ALZ-22-e71742-s005.docx]

## Supplemental Figure 1. Quality control metrics for single-nucleus RNA-seq data.

Violin plots displaying (A) the number of unique genes detected per nucleus (nFeature_RNA), (B) the total number of detected transcripts per nucleus (nCount_RNA), and (C) the percentage of mitochondrial transcripts (percent.mt), across genotypes. Nuclei with >1% mitochondrial content were filtered out prior to analysis. Red lines indicate median values.

## Supplemental Figure 2.

(A) Dotplot of module expression across clusters. Dot color indicates the module's average scaled expression (blue = low, red = high); dot size represents the percentage of nuclei within that cell type expressing the module.

(B-C) Enrichment analysis of modules 5 (B) and 7 (C), based on GO Biological Process. Bar colors correspond to the module colors shown in the feature plot in the main Figure. 2A, with the gradient indicating the −log10 of the Benjamini–Hochberg adjusted p-value. The combined score integrates the significance of gene set overlap (log p) with the deviation from expected rank (z-score) for each term in the gene set library.

## Supplemental Figure 3.

(A) Bar plot displaying the number of 5XFAD;PS19-exclusive DEGs per 1,000 nuclei in each cell type.

(B-F) UpSet plots showing the number of differentially expressed genes (set size) in each group versus wild type and their overlap across comparisons for the representative clusters of oligodendrocytes (O_1) (B), excitatory neurons (ExN_1) (C), astrocytes (A_1) (D), endothelial cells (EC_1) (E), and fibroblasts (FB) (F).

(G-K) Enrichment analysis of all 5XFAD;PS19 DEGs in the same clusters (O_1, G; ExN_1, H; A_1, I; EC_1, J; FB, K) based on GO Biological Process 2023 and filtered for significant terms (-log10 adjusted p-value > 1.3). The bar color reflects -log10 of Benjamini–Hochberg adjusted p-value. The combined score integrates the significance of gene set overlap (log-transformed p-value from Fisher’s exact test) with the deviation from expected rank (z-score) for each term in the gene set library.

## Supplemental Figure 4.

(A-B) Stacked violin plots showing scaled expression of marker genes for the disease-associated oligodendrocytes (DAOs) for oligodendrocyte clusters O_1, O_2, and O_3 (A) and by genotype for the same clusters (B).

(C-E) Stacked violin plot showing scaled expression of marker genes for the disease-associated microglia (DAM)-like (C), antigen presentation (HLA/MHC class II) (D), and cytokine response (E) microglial states by genotype for clusters M_1, and M_2.

(F-G) Heatmaps showing per-gene z-scores of all DEGs contributing to enriched terms in the individual samples of WT (S1-S4) versus 5XFAD;PS19 (S5-S8) in M_2 (F) and O_3 (G). Z-scores are color-coded from blue (below mean), through white (mean), to red (above mean).

## Supplemental Figure 5.

(A-B) Alluvial plots illustrating outgoing (A) and incoming (B) communication patterns in 5XFAD;PS19, with corresponding clusters (left panel) and signaling pathways (right panel) in coordination. The thickness of the flow indicates the contribution of the cluster to the corresponding communication pattern.

(C) Pathway-level information flow was analyzed using a paired Wilcoxon test to assess whether signaling flow differed significantly between WT and 5XFAD;PS19. Pathways enriched in WT are shown in red, while those enriched in 5XFAD;PS19 are shown in blue. PROS and VISTA are highlighted in yellow.

## Supplemental Figure 6.

(A-B) Heatmaps of PROS (A) and VISTA (B) pathways showing the major contributors to outgoing (rows) or incoming (columns) signaling across clusters in WT (left) and 5XFAD;PS19 (right). The heatmap color scale represents communication probability, i.e., the relative signaling strength of a pathway across clusters. The bar plot summarizes the total signaling strength per cluster by aggregating outgoing (row) or incoming (column) signaling.

## Supplemental Figure 7.

(A) NES across different regions of AMP-AD human datasets (ROSMAP – Dorolateral prefrontal cortex, MSSM – Frontal Pole, MSSM – Superior Temporal Gyrus, MSSM – parahippocampal gyrus, Mayo Clinic – cerebellum, Mayo Clinic – temporal cortex). GO terms identified by GSEA are grouped into AD biodomains. Dot size reflects -log10(adjusted p-value) for each GO term.

(B) Bar graph displaying the number of enrichment terms in each mouse model that are concordant with human AMP-AD data, grouped by biodomain.
